# SARS-CoV-2 infection in an infant with non-respiratory manifestations: a case report

**DOI:** 10.1186/s43054-020-00047-7

**Published:** 2021-01-25

**Authors:** Muhammad Adel, Ahmed Magdy

**Affiliations:** 1 Pediatric Department, Faculty of Medicine, Fayoum University Children’s Hospital, Fayoum, Egypt; 2 Radiology Department, Faculty of Medicine, Fayoum University Hospital, Fayoum, Egypt

**Keywords:** COVID-19, Pediatrics, Pancytopenia, Case report

## Abstract

**Background:**

Coronavirus disease (COVID-19) presents in children usually with less severe manifestations than in adults. Although fever and cough were reported as the most common symptoms, children can have non-specific symptoms. We describe an infant with aplastic anemia as the main manifestation.

**Case presentation:**

We describe a case of SARS-CoV-2 infection in an infant without any respiratory symptoms or signs while manifesting principally with pallor and purpura. Pancytopenia with reticulocytopenia was the predominant feature in the initial laboratory investigations, pointing to aplastic anemia. Chest computed tomography surprisingly showed typical findings suggestive of SARS-CoV-2 infection. Infection was later confirmed by positive real-time reverse transcription polymerase chain reaction assay (RT-PCR) for SARS-CoV-2.

**Conclusions:**

Infants with COVID-19 can have non-specific manifestations and a high index of suspicion should be kept in mind especially in regions with a high incidence of the disease. Chest computed tomography (CT) and testing for SARS-CoV-2 infection by RT-PCR may be considered even in the absence of respiratory manifestations.

## Background

The new coronavirus was first reported in Wuhan, China, in December 2019, as the cause of severe pneumonia [1]. The virus is named SARS-CoV-2 and causes a disease called COVID-19 [2]. In March 2020, the WHO has declared it as a pandemic [3]. Although COVID-19 can affect all age groups, it presents in children usually with milder manifestations than in adults and may cause non-specific symptoms [4]. However, infants, in particular, were found to be vulnerable to SARS-CoV-2 infection [5]. In China, a cohort of 171 confirmed COVID-19 cases in children revealed that fever, cough, and pharyngeal erythema were the most common manifestations [6]. In Italy, The Coronavirus Infection in Pediatric Emergency Departments (CONFIDENCE) study [7] that involved 100 Italian children with COVID-19 reported similar results with fever and cough being the most common symptoms.

None of the cases described by the CONFIDENCE study [7] nor by Lu et al. [6] were reported to have pancytopenia. A cytokine profile resembling secondary hemophagocytic lymphohistiocytosis (sHLH) is found to be associated with some cases of COVID-19 [8]. So, sHLH could be triggered by COVID-19. We describe here a COVID-19 case of an infant presenting with pallor and purpura as the main symptoms without respiratory symptoms.

## Case presentation

**Fig. 1:**
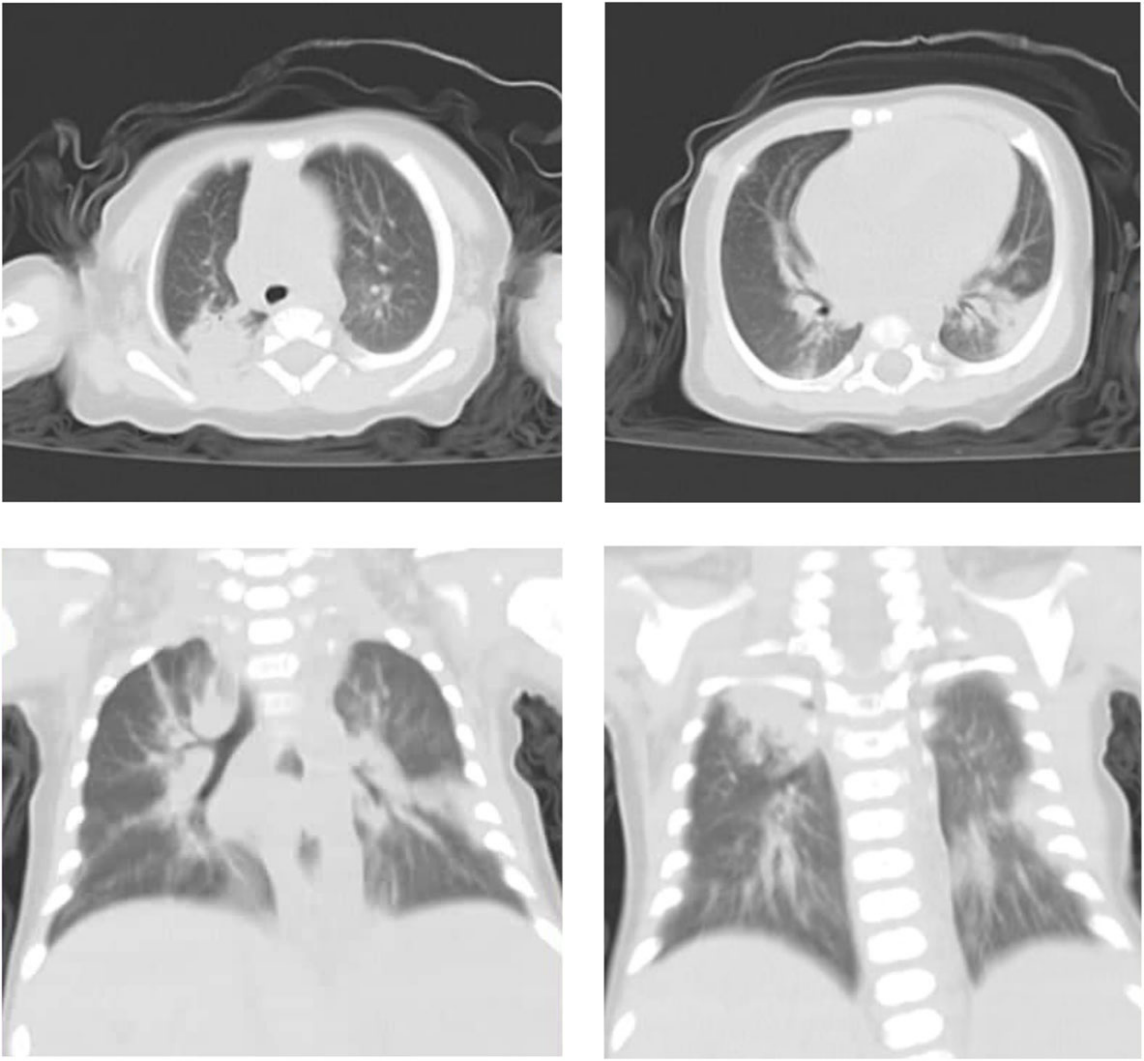
Chest CT findings

The patient is a 71-day-old, full-term, previously healthy male infant. Before admission, he developed a low-grade fever and two vomiting episodes for 2 days. He received paracetamol as outpatient then fever and vomiting subsided. No other drugs were taken. Three days later, he developed acute onset of pallor and purpura which were the principal presentations to the emergency department on 28 April 2020. No respiratory symptoms or any other symptoms were reported. His general condition was good. He had sinus tachycardia with a heart rate of 176/min but with normal respiratory rate, capillary refill time, blood pressure, and oxygen saturation. He had no dysmorphic features or any skeletal anomalies. His chest examination was normal with no respiratory distress. However, hepatosplenomegaly was detected. No focal signs of infection were found. His initial laboratory testing (Table 1) revealed marked pancytopenia with low reticulocytic count (0.2%). The direct blood film showed no abnormal cells. A bone marrow aspirate was performed and found to be hypocellular without abnormal cells. Other tests showed coagulopathy, mildly elevated liver enzymes, markedly elevated ferritin, elevated triglycerides. Kidney function tests were normal and blood and urine cultures were negative. Bone marrow biopsy or liver biopsy were not done. Patient received supportive therapy with transfusion of packed RBCs for three times to correct his anemia, and he also received fresh frozen plasma for three times to correct coagulopathy. He was admitted for 6 days at pediatric ward. During admission, he had fever for 2 days. However, there were no cough, pharyngeal erythema, or any other respiratory symptoms or signs all through during the admission period. Vital signs were normal without any deterioration in his general condition. Although the patient had no respiratory symptoms or signs of COVID-19, a chest computed tomography (CT) was performed particularly in the setting of increasing incidence of COVID-19 in Egypt at that time. It was done on day 4 of admission which surprisingly revealed (Fig. 1) multiple scattered consolidative patches scattered at both lung fields, being distributed mainly posteriorly, implicating posterior and apico-posterior segments of right upper lobe, lateral lingular segment, and to less extent, the lateral basal segment of left lower lobe. Mild left pleural effusion was also noted. CO-RADS is a radiological classification for the possibility of COVID 19 with the level of suspicion increasing from very low (CO-RADS 1) to very high (CO-RADS 5) [9]. RT-PCR-proven SARS-CoV-2 infection at the time of examination is classified as category CO-RADS 6 [9]. The patient had category CO-RADS 5 based on CT findings only. The infant was immediately isolated and tested for SARS-CoV-2 by RT-PCR of nasopharyngeal swab that was surprisingly positive, confirming the infection and putting him in the category CO-RADS 6. Patient was then referred to an isolation hospital for COVID-19 cases. He developed vasoplegic shock and died.

**Table 1:** Laboratory tests

	28/4 morning sample	28/4 Evening sample	29/4	1/5	3/5
Hemoglobin (g/dL)	2.9	2.8	6.3 After PRBCs transfusion		10.5 After PRBCs transfusion
MCV (fL)	81	88.9			78.7
MCH (pg)	26.4	31.1			26.6
Corrected reticulocytic count %	0.4%	0.2%			
White blood cell count (× 10^9^/L)	2.7	4.94	4.19		2.39
Lymphocytic count (× 10^9^/L)	2.29 (85%)	4.25 (86%)			
Neutrophil count (× 10^9^/L)	0.134 (5%)	0.197 (4%)			
Monocyte count (× 10^9^/L)	0.27 (10%)	0.494 (10%)			
Platelet count (× 10^9^/L)	40	36	44		5
Bone marrow aspirate			Hypocellular		
C-reactive protein (mg/L)	3				6
Erythrocyte sedimentation rate (mm/h)	16				
Ferritin (ng/mL)				5983	
Triglycerides (mmol/L)				5.6	
Alanine aminotransferase (U/L)	73		79		34
Aspartate aminotransferase (U/L)	96		110		44
International normalized ratio (INR)			4.13	1.1	1.2
Albumin (g/L)	38				39
EBV VCA IgM			Negative		
CMV IgM			Negative		
Blood culture	Obtained			No growth	
pH	7.54				
pCO_2_ (mmHg)	26				
HCO_3_ (mmol/L)	24.7				
RT-PCR for SARS-CoV-2				Obtained	Positive

*Abbreviations*: *MCV* mean corpuscular volume, *MCH* mean corpuscular hemoglobin. *EBV VCA* Epstein-Barr virus-viral capsid antigen antibody, *CMV* cytomegalovirus, *PRBCs* packed red blood cells

## Discussion

This report describes an interesting case of SARS-CoV-2 infection that presented with fever, pallor, and purpura as the initial presentation in the absence of any respiratory symptoms or signs. COVID-19 presents in children usually with milder manifestations than in adults [4]. However, young children, especially infants, were found to be vulnerable to SARS-CoV-2 infection with a higher proportion of severe cases than any other pediatric age groups [5]. COVID-19 most commonly presents with a spectrum of signs and symptoms ranging from completely asymptomatic to symptoms of acute upper respiratory tract infection such as fever, cough, sore throat, rhinorrhea, and shortness of breath. In more severe cases, patients can have respiratory failure, shock, coagulopathy, and renal injury [5]. Regarding our patient’s clinical presentation, it was non-specific, having no respiratory symptoms or signs all during the period of hospitalization. However, this case had principally features of aplastic anemia with acute onset of pallor and purpura following fever and vomiting. Cai et al. [10] described five cases of COVID-19 in children that also presented non-respiratory manifestations as the first manifestation.

The finding of pancytopenia with reticulocytopenia and hypocellular bone marrow in this case was the guide toward a diagnosis of aplastic anemia (AA). Drug intake, viral infection, HLH, malignancy, bone marrow infiltration by a storage disorder, and inherited bone marrow failure syndromes (IBMFS) were the main suspected causes of AA in this case. Serology markers of Epstein-Barr virus (EBV) and cytomegalovirus (CMV) were negative. There was no history of drug intake prior to the presentation apart from paracetamol. Similarly, there were no features to suggest IBMFS (as there were no any developmental delay, family history of cytopenias or congenital anomalies, osteopetrosis, short stature, limb anomalies, or any other anomalies). Features suggestive of HLH in this patient were markedly elevated ferritin, high triglycerides, and pancytopenia in addition to clinically detected hepatosplenomegaly and fever. So, a diagnosis of AA secondary to HLH was considered.

Neither the Italian CONFIDENCE study [7] nor Lu et al. [6] in China reported COVID-19 cases with pancytopenia. Both studies reported only leukopenia and/or thrombocytopenia in some cases. A cytokine profile resembling sHLH is found to be associated with COVID-19 in some cases which is characterized by increased interleukin (IL)-2, IL-7, granulocyte colony stimulating factor, interferon-γ inducible protein 10, and many other factors [8]. This may indicate that sHLH could be triggered by COVID-19. It was reported that viral infections may trigger secondary forms of HLH [11]. Pancytopenia represents a great challenge for the diagnosis of COVID-19 because the clinical manifestations of respiratory affection were absent.

As regards the CT chest findings, this case report suggests that infants with SARS-CoV-2 infection may not exhibit respiratory symptoms and signs even in the presence of radiological findings of pneumonia. Similarly, Lu et al. [6] reported that out of 171 children with confirmed SARS-CoV-2 infection, only 12 had radiologic findings of pneumonia without having any symptoms of infection. Cai et al. [10] also reported five children with COVID-19 who had radiological findings of pneumonia in chest CT without having any respiratory symptoms or signs.

This case highlights that aplastic anemia due to sHLH can be the initial and the principal presenting feature of COVID-19. Reliance on respiratory symptoms and signs only for testing will easily miss cases in whom these symptoms and signs are absent while fever alone or symptoms of other organ system affection are the first presenting features. As the predominant clinical manifestations of COVID-19 can vary from one patient to another, one should keep a high index of suspicion, especially in the setting of increasing incidence of COVID-19 cases.

## Conclusion

SARS-CoV-2 infection in infants should be kept in mind in those presenting with non-specific clinical picture such as fever and vomiting, especially in epidemic areas. Chest CT, in addition to nucleic acid testing for SARS-CoV-2, may be considered for diagnosing cases regardless of the presence or absence of respiratory symptoms or signs, particularly in regions with increasing incidence of COVID-19 cases. We are still in need of further reporting of the clinical presentation pattern of COVID-19 cases in children.

## Data Availability

Not applicable.

## Abbreviations

SARS-CoV-2: Severe acute respiratory syndrome coronavirus 2
COVID-19: Coronavirus disease 2019
WHO: World Health Organization
sHLH: Secondary hemophagocytic lymphohistiocytosis
HLH: Hemophagocytic lymphohistiocytosis
CT: Computed tomography
RT-PCR: Real-time reverse transcription polymerase chain reaction assay
AA: Aplastic anemia
IL: Interleukin
EBV: Epstein-Barr virus
CMV: Cytomegalovirus
